# Neurometabolite Levels in Alcohol Use Disorder Patients During Baclofen Treatment and Prediction of Relapse to Heavy Drinking

**DOI:** 10.3389/fpsyt.2018.00412

**Published:** 2018-09-04

**Authors:** Kirsten C. Morley, Jim Lagopoulos, Warren Logge, Kate Chitty, Andrew Baillie, Paul S. Haber

**Affiliations:** ^1^NHMRC Centre of Research Excellence in Mental Health and Substance Use, Central Clinical School, Sydney Medical School, University of Sydney, Sydney, NSW, Australia; ^2^Sunshine Coast Mind and Neuroscience, University of Sunshine Coast, Birtinya, QLD, Australia; ^3^School of Pharmacology, Sydney Medical School, University of Sydney, Sydney, NSW, Australia; ^4^NHMRC Centre of Research Excellence in Mental Health and Substance Use, Health Sciences, University of Sydney, Sydney, NSW, Australia; ^5^Drug Health Services, Royal Prince Alfred Hospital, Sydney, NSW, Australia

**Keywords:** GABA, glutamate, GSH, NAA, baclofen, alcohol dependence

## Abstract

**Background and Aims:** Baclofen, a GABA_B_ agonist, is used as a treatment for alcohol dependence. We aimed to examine brain metabolites following administration of baclofen or placebo in alcohol dependent individuals enrolled in a randomized placebo-controlled trial.

**Methods:** Participants included 31 alcohol dependent individuals (recent drinking: *N* = 16; and abstinent: *N* = 15) who had received daily baclofen (BAC 30–75 mg = 20) or placebo (PL = 11) for at least 2 weeks (average 17 days). Using *in vivo* proton magnetic resonance spectroscopy (^1^H-MRS), spectra from the right parietal lobe were analyzed to obtain measures of GABA, Glutamate (Glu), Glutathione (GSH) and N-Acetyl Apartate (NAA) 120 min following administration of PL or BAC.

**Results:** When weighting alcohol dependent participants according to recent alcohol consumption (within 24 h), there were significant differences between BAC and PL on parietal concentrations of GSH (*p* < 0.01) and NAA (*p* < 0.05). Multiple linear regression revealed a significant predictive effect of GSH on heavy drinking days at 12 weeks follow-up (Model: *F* = 14.28, *R*^2^ = 0.85; GSH: *B* = −1.22, *p* = 0.01) and also percentage days abstinent at 12 weeks follow-up (Model: *F* = 6.50, *R*^2^ = 0.72; GSH: *B* = 0.99, *p* = 0.06).

**Conclusion:** Our data provide preliminary evidence that the effect of baclofen may be mediated by increased parietal concentrations of the antioxidant GSH and NAA in recently drinking alcohol dependent patients. GSH/Cr levels were also predictive of improved drinking outcomes in the trial and suggests a role for neural oxidative stress in alcohol use disorder.

## Introduction

Alcohol dependence is a common disorder characterized most often by chronic relapses to heavy alcohol consumption (1). Although alcohol use disorders are leading causes of preventable death treatment options are still limited. Heavy chronic alcohol use can result in a down-regulation of ɤ-aminobutyric (GABA) receptor activity, dysregulation of glutamatergic neurotransmission and disinhibition of the reward pathway. Baclofen, a selective GABA_B_ receptor agonist, has emerged as a potential treatment for alcohol dependence and is thought to counterbalance these processes given that presynaptic GABA_B_ receptors regulate neuronal excitability and neurotransmitter release in many different neuronal pathways including the mesolimbic system (2, 3).

While there has been expanded utilization of baclofen in the treatment of alcohol dependence (4), there remains controversy in the field with mixed results from clinical trials [for example see (5–11)]. We have recently demonstrated that baclofen is effective in increasing abstinence in patients with and without alcoholic liver disease (12). There is significant variation in treatment response and not all individuals with alcohol dependence respond favorably to baclofen. Alcohol dependence is a complex and heterogeneous disorder involving disruption of multiple neurobiological mechanisms whereby advances in the understanding of this heterogeneity and associated variations in response to pharmacotherapies will have clinical appeal for treatment.

Proton magnetic resonance spectroscopy (^1^H-MRS) enables the *in vivo* detection of neurometabolites whose levels may be reflective of neurobiological dysregulation associated with chronic alcohol use disorders in humans (13). Correspondingly, ^1^H-MRS enables the investigation of the effects of pharmacological treatment such as baclofen on these abnormalities. There are some inconsistencies in the literature with regards to the levels of metabolite concentrations in patients with alcohol use disorder yet a substantial proportion of this variation can be explained by differences in recent alcohol consumption. ^1^H-MRS studies have generally demonstrated brain Glu levels in alcohol patients relative to healthy non-drinking controls to be significantly lower during intoxication (14), higher during initial withdrawal (15) and low again at least 7 days from last alcohol consumption (16). Glutathione (GSH) is a key antioxidant synthesized in cells and has been found to be reduced in the hippocampus in heavy drinkers (17). Reduced levels of N-acetylaspartate (NAA), a marker of neuronal integrity, have also been reported in frontal lobes (18) and these have been found to negatively correlate with recent heavy alcohol consumption (13). There have been a paucity of ^1^H-MRS studies examining GABA concentrations in patients with alcohol use disorder with some research reporting decreased GABA (19) while other studies have not been able to replicate these findings (16).

Many of these neurochemical aberrations reported above at least partially normalize following prolonged abstinence (between 14 and 35 days) (15, 16). This early time period of recovery is particularly challenging for many patients. ^1^H-MRS investigations of the effects of alcohol pharmacotherapies on metabolite concentrations have revealed significant amelioration of these abnormalities following treatment relative to placebo. For example, two studies have reported that acamprosate reduced glutamate concentrations in the anterior cingulate cortex (20, 21). To date, there have been no ^1^H-MRS studies examining neurometabolite concentrations following baclofen administration in alcohol patients or other clinical populations.

The present study therefore sought to investigate (i) neurometabolite levels following administration of baclofen (BAC) or placebo (PL); and (ii) the relationship between neurometabolite ratios (glutamate/Cr, GSH/Cr, NAA/Cr) and GABA^+^, and future drinking outcomes previously defined (as heavy drinking days, percentage abstinent days and drinks per drinking days) from a randomized controlled trial of BAC vs. PL (12).

## Methods

The study was approved by the Human Ethics Review Committee of the Sydney Local Health District (X11-0154). The study involved off-label use of a registered medication in Australia and approval was given under the Clinical Trial Notification (CTN) scheme of the Therapeutics Goods Administration (TGA) (2013/0060) as part of a clinical trial (ClinicalTrials.gov, NCT01711125, https://clinicaltrials.gov/ct2/show/NCT01711125). All participants included in this MRS substudy provided written informed consent after commencement of randomization for the main trial.

### Participants

Participants were recruited from a larger clinical trial investigating baclofen in the treatment of alcohol dependence. The main study rationale, design, and methods for the clinical trial with which these patients were recruited have been previously detailed (22) and the results reported (12). Eligibility included: (i) alcohol dependence according to the ICD-10 criteria; (ii) age 18–75; (iii) adequate cognition and English language skills to give valid consent and complete research interviews; (iv) willingness to give written informed consent; (v) abstinence from alcohol for between 3 and 21 days leading up to randomization; (vi) resolution of any clinically evident alcohol withdrawal (CIWA-AR); (vii) <48 h after ceasing any diazepam required for withdrawal management.

Exclusion criteria: (i) active major mental disorder associated with psychosis or significant suicide risk, (ii) pregnancy or lactation, (iii) concurrent use of any psychotropic medication other than antidepressants (provided these are taken at stable doses for at least 2 months); (iv) unstable substance use; (v) clinical evidence of persisting hepatic encephalopathy (drowsiness, sleep inversion or asterixis); (vi) pending incarceration; (vii) lack of stable housing, (viii) peptic ulcer; (ix) unstable diabetes mellitus.

### Procedure

For the main trial, participants received upward and downward titrations of medication for the 84 days of treatment and were randomized 1:1:1 to baclofen took a capsule of 10 or 25 mg: 1 × day for the first 2 days, 2 × day on days 3–4, 3 × day on days 5–80, 2 × day on days 81–82 and finally 1 × day for the last 2 days. The PL pills, which were identical in appearance, were also titrated upward and downward to maintain the double blind. All subjects received one medical and research assessment and five follow-up reviews over the 12-weeks treatment period. Participants underwent ^1^H-MRS scanning at week 2 (on average 17 days following enrolment), approximately 120 min post-administration of either 10, 25 mg BAC or PL. Breath alcohol concentration was obtained and only participants with a reading of 0.00 were permitted to proceed with the scan. Participants were informed to abstain from caffeine for 4 h prior to scan session.

### Assessments

A detail list of assessments has been outlined in detail previously (22). At baseline, structured diagnostic information regarding alcohol dependence and demographic variables were gathered. Alcohol consumption in the previous 30 days was determined using the timeline follow back (TLFB) alcohol consumption form (23) and a daily monitoring diary utilized in our previous alcohol treatment studies (8, 24, 25). Severity of alcohol dependence was assessed using the Alcohol Dependence Scale (ADS) (26), craving was measured by the Penn Alcohol Craving Scale [PACS; (27)]. In addition, trained interviewers conducted a structured psychiatric diagnostic interview using the Mini International Neuropsychiatric Interview (M.I.N.I.) (28). The treatment outcomes for this study were derived from drinking measures [TLFB] obtained during each visit.

### ^1^H-MRS

**Figure 1:**
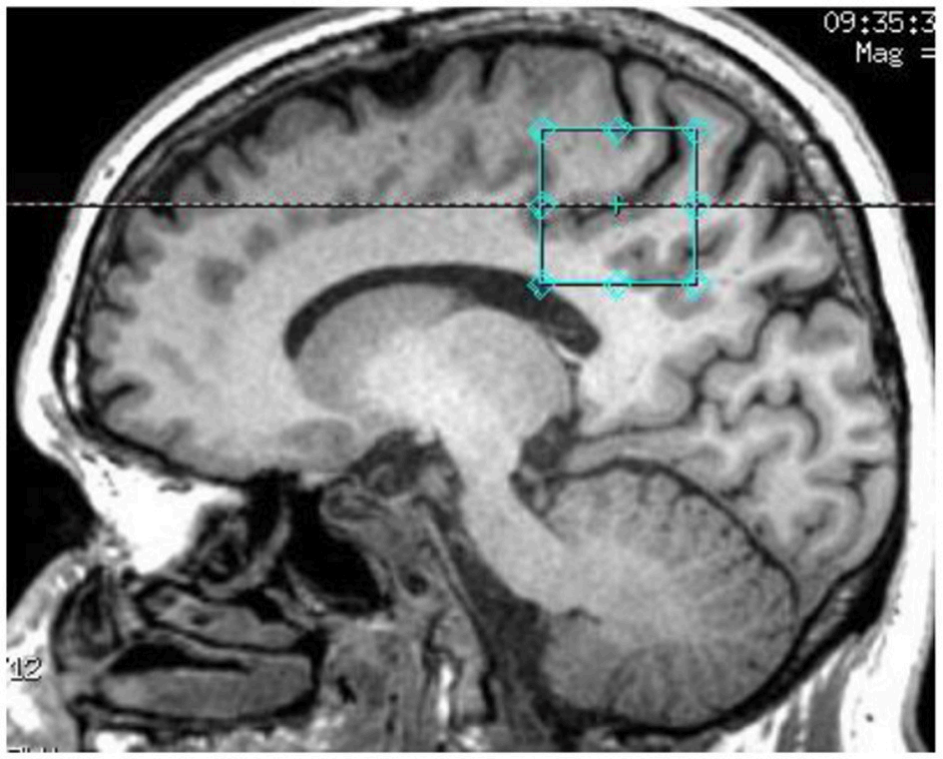
T1-weighted structural image showing the anatomical localization of 3 × 3 × 3 acquisition voxel.

^1^H-MRS spectra were acquired on a 3 Tesla GE Discovery MR750 scanner using an 8-channel phased array headcoil. The protocol comprised a three-dimensional sagittal whole-brain scout for orientation and positioning of all subsequent scans (TR = 50 ms; TE = 4 ms; 256 matrix; no averaging, *z* = 5 mm thickness). To aid in the anatomical localization of the sampled voxels as well as gray–white-CSF segmentation, the T1-weighted structural image acquired at the beginning of the scan session was used (MPRAGE sequence: TR = 7.2 ms; TE = 2.8 ms; flip angle = 10°; matrix 256 × 256; 0.9 mm isotropic voxels, 196 slices). Next, a single voxel PRESS acquisition with two chemical shift-selective imaging pulses for water suppression (TR = 2,000 ms; TE = 35 ms; 128 averages) was acquired for GSH, Glu, NAA and Cr. Single voxel MEGA-PRESS (29) was acquired for GABA^+^ determination [TR = 1,800 ms; TE = 68 ms; NEX (phase cycling) = 8; number of acquisitions = 256; number of points = 4,096; spectral width = 5,000]. Anatomical localization of the voxel placement in the right parietal lobe (voxel size = 3 × 3 × 3 cm^3^) was based on the Talairach and Tournoux brain atlas and positioning was guided by the T1-weighted image (see Figure 1). Unsuppressed water scans (acquired from the same voxel) were collected prior to acquisition of the metabolite scans. All spectra were shimmed to achieve line widths (FWHM) of <15 Hz. Prior to any post-processing all spectra were visually inspected separately by two independent raters, to ensure the consistency of the data. Poorly fitted metabolite peaks, as reflected by large Cramer–Rao Lower Bounds (CRLB) (i.e., >20%) were excluded from further analysis.

**Figure 2:**
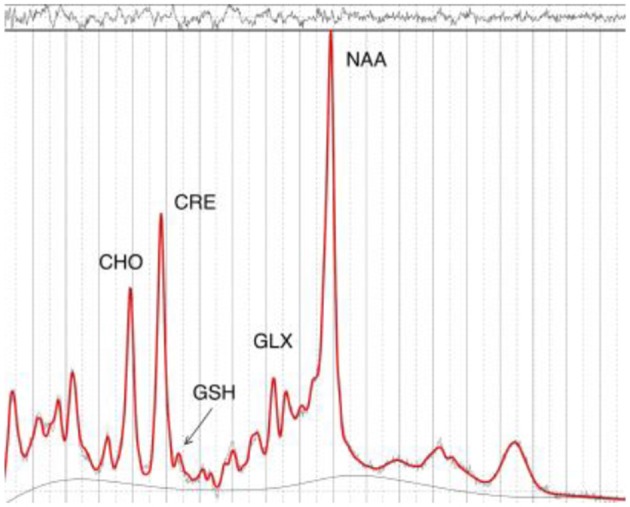
Example MR-spectrum from the right parietal cortex.

Following ^1^H-MRS acquisition, data were transferred offline for post-processing. GABA data were processed using the Gannet software toolkit (30). In brief, the data were first processed using the GannetLoad module which parses variables from the data headers and applies a line broadening of 3 Hz. Next, individual spectra were frequency and phase corrected using Spectral Registration. The data were then processed by the GannetFit module, which employs a single Gaussian model to fit the edited GABA^+^ signal and evaluates GABA concentration in institutional units relative to water. The quality of the data were determined by the overall “Fit Error” index of each subject. This index represents the standard deviation of the fitting residual divided by the amplitude of the fitted peaks, and thus a measure of the signal-to-noise ratio. Only spectra with a relative Fit Error of GABA^+^ below 10% were used for the subsequent statistical analyses. Next, the LCModel software package (31) was used to estimate GSH, Glu, NAA, and Cr (see Figure 2). The radiographers and the neuroimaging expert who read the spectroscopy data were blinded to group allocation.

### Statistical analysis

Although participants were randomized to medication allocation, only consenting individuals participated in the neuroimaging arm of the study. Thus, baseline variables were examined for differences between groups to examine differences between the groups on continuous baseline demographic and clinical characteristics (ANOVA) and χ^2^ tests were performed for categorical variables.

As previously outlined in the original protocol paper (22) and the main effects analysis from the trial (12), planned analyses were conducted including PL (placebo) vs. BAC (baclofen: composite of the two doses). One way ANCOVA were used to examine medication differences for the neurometabolites (Glu/Cr, GSH/Cr, GABA^+^, NAA/Cr) between BAC and PL with recent drinking (24 h) placed as covariates. For neurometabolites that showed significant differences, we then performed exploratory analyses across all three treatment group (separating the doses) followed by *post-hoc* tests between each of the two doses (30 vs. 75 mg) and PL. We also explored the role of time on treatment with BAC (days) on metabolite correlations with bivariate correlations. We also used bivariate correlations to examine the association of metabolite concentrations following BAC vs. PL administration and later drinking outcomes on the trial. We then placed relevant metabolites into a linear regression weighting for recent alcohol consumption (previous 24 h). Drinking outcomes, as outlined in the main trial (12) and previous protocol (22), included percentage days abstinent and heavy drinking days (although both variables were calculated from the day after the scan until the end of the trial).

All analyses were two-tailed, with significance level at *P* < 0.05. Data were analyzed using SPSS 23 for Mac OSX.

## Results

### Sample characteristics

Of the 31 patients recruited for the neuroimaging study, 9 were randomized to receive placebo, 11 to receive baclofen 30 mg and 11 to receive baclofen 75 mg. As per our main results paper groups were analyzed as placebo vs. baclofen (composite doses). Baseline characteristics are displayed in Table 1. There were no significant differences between treatment groups with regards to sociodemographic or clinical characteristics (*F*s < 1.99, *p's* > 0.17 for continuous and χ^2^'s < 0.22, *p's* > 0.26 for categorical).

**Table 1:** Demographic and clinical characteristics of participants.

**Characteristic**	**Placebo** **(*n* = 11)**	**Baclofen 30–75 mg** **(*n* = 20)**
Age, y	51.73 ± 12.06	48.65 ± 9.24
Gender, % *F*	46	25
Education, y	14.36 ± 2.63	14.45 ± 2.38
Unemployed, %	18	40
Drinks per drinking day^a^	12.12 ± 4.34	9.92 ± 5.90
Years since alcohol-related problems began	14.15 ± 9.59	16.23 ± 11.24
Alcohol dependence severity	13.73 ± 6.18	16.95 ± 8.29
PACS craving	17.91 ± 5.11	15.60 ± 5.62
Alcoholic liver disease, %	27	20
Cigarette smokers, %	73	75
Antidepressant use, %	82	60

Data represent mean ± SD unless otherwise noted.

a *During the 30 days preceding the first day of the study, based on the Time-Line Follow-Back method. ADS, Alcohol Dependence Severity Scale; PACS, Penn Alcohol Craving Scale. There were no significant differences between groups on baseline variables*.

### Metabolite concentrations following acute administration of BAC vs. PL

**Figure 3:**
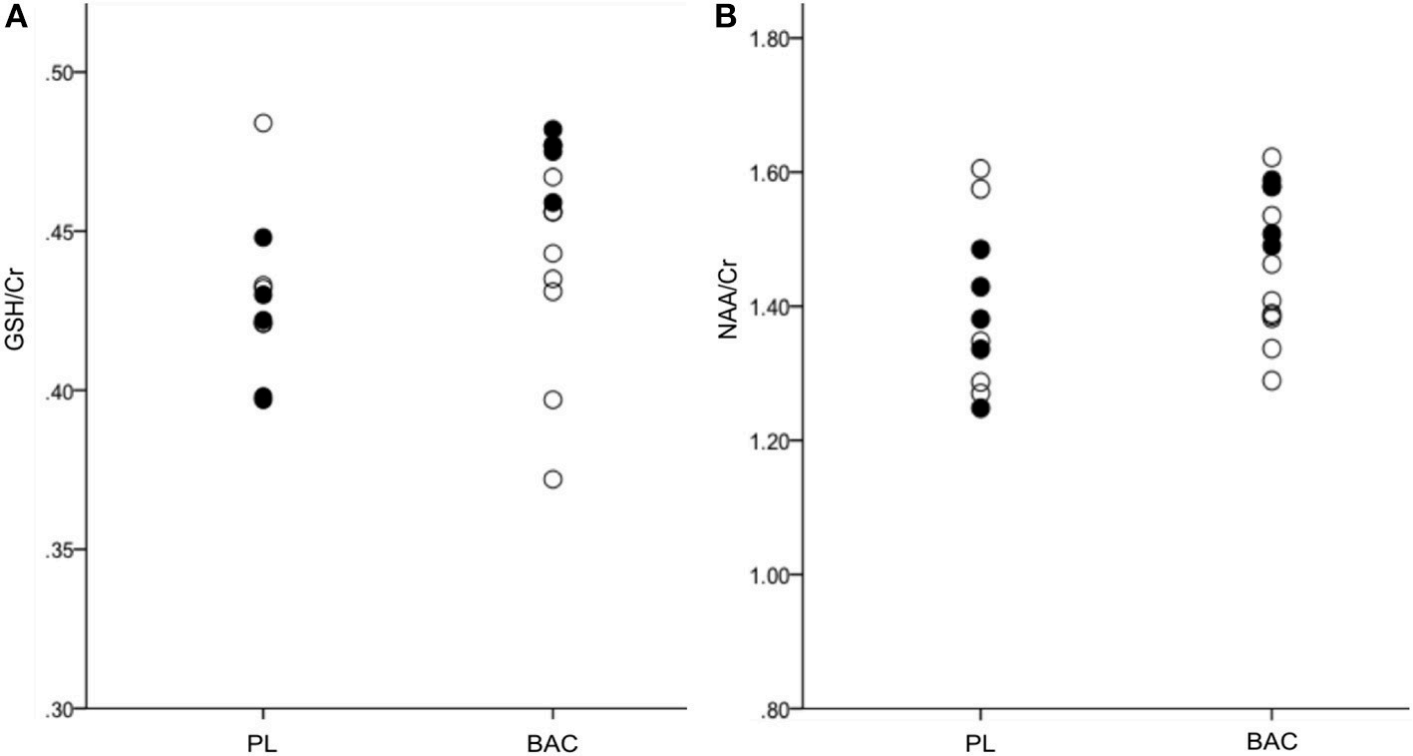
**(A)** GSH/Cr and **(B)** NAA/Cr in the right parietal cortex for PL (placebo) and BAC (baclofen: 30–75 mg) treated alcohol dependent patients (•, recent alcohol consumption <24 h; °, no recent alcohol consumption <24 h).

Means for each of the metabolites following administration with BAC or PL (as per alcohol consumed within the previous 24 h) are presented in Table 2. There were no significant differences between groups (PL vs. BL) for any of the metabolite concentrations (GABA^+^: *F* = 0.35, *p* = 0.56; Glu/Cr: *F* = 0.50, *p* = 0.49; GSH/Cr: *F* = 2.46, *p* = 0.13; NAA/Cr: *F* = 2.34, *p* = 0.14). However, when weighting alcohol dependent participants according to recent drinking (24 h) there were significant differences between BAC and PL on GSH/Cr (*F* = 20.88, *p* < 0.01) and NAA/Cr (*F* = 10.63, *p* < 0.05; see Figure 3). There were no significant differences between BAC and PL for the other metabolite concentrations (GABA^+^: *F* = 3.70, *p* = 0.10; Glu/Cr: *F* = 2.81, *p* = 0.14).

**Table 2:** ^1^H-MRS neurometabolite concentrations of participants treated with either baclofen (30–75 mg dose) or placebo.

**Metabolites**	**Placebo**	**Baclofen 30–75 mg**
Full sample, *n*	11	20
GABA^+^	0.55 ± 0.04	0.53 ± 0.09
Glu/Cr	1.50 ± 0.07	1.47 ± 0.09
GSH/Cr^*^	0.43 ± 0.03	0.45 ± 0.03
NAA/Cr^*^	1.40 ± 0.13	1.47 ± 0.11
NAAG/Cr	0.14 ± 0.05	0.13 ± 0.07
Recent alcohol consumption^∧^, *n* (%)	5 (46)	4 (20)
GABA^+^	0.53 ± 0.04	0.59 ± 0.05
Glu/Cr	1.47 ± 0.06	1.55 ± 0.08
GSH/Cr^*^	0.42 ± 0.02	0.47 ± 0.01
NAA/Cr^*^	1.38 ± 0.09	1.54 ± 0.05
NAAG/Cr	0.12 ± 0.03	0.09 ± 0.05

Data represent raw means ± SD.

∧ in previous 24 h before scanning, Y/N.

* *p < 0.05, comparing baclofen (30–75 mg) vs. placebo. Patients were previously treated with either BAC or PL for an average of 17 days and were scanned approximately 120 min following administration of BAC or PL*.

### Association of metabolite concentrations with previous treatment days with BAC

There were significant correlations between previous treatment days with BAC and GSH/Cr (*r* = 0.44, *p* = 0.03) and NAA/Cr (*r* = 0.43, *p* = 0.04). When examining these associations in only the BAC-treated patients there was a trend for significance for GSH/Cr (*r* = 0.48, *p* = 0.10) and the association between NAA/Cr and days treated with BAC remained significant (*r* = 0.54, *p* = 0.05). There were no significant correlations between other metabolite concentrations and previous treatment days with BAC (entire sample: *p*'s > 0.52; only BAC-treated patients: *p* >'s 0.14).

### Association of metabolite concentrations with drinking outcomes at follow-up

There was a significant negative correlation between Heavy Drinking Days (at follow-up) and GSH/Cr (*r* = −0.47, *p* = 0.04). There were no significant correlations between metabolite concentrations and other drinking outcomes (*p*'s > 0.10). The results of the multiple linear regression are presented in Table 3. We entered relevant neurometabolites (significant in the acute administration study and bivariate associations: GSH, NAA) into a multiple regression (Model 1) and then weighted for recent alcohol consumption (Model 2). For heavy drinking days, there was no significant effect of the model (*F* = 2.61, *R*^2^ = 0.24, *p* = 0.10). When examining only patients that reported recent alcohol consumption (within the last 24 h) the model was significant (*F* = 14.28, *R*^2^ = 0.85^**^ *R*^2^ change = 0.61, *p* = 0.01) with a significant predicting effect of GSH (*B* = −1.22, *p* = 0.01). For percentage days abstinent, there was no significant effect of the model (*F* = 0.49, *R*^2^ = 0.10, *p* = 0.62). When examining only patients that reported recent alcohol consumption (within the last 24 h) the model was significant (*F* = 6.50, *R*^2^ = 0.72^**^ *R*^2^ change = 0.62, *p* = 0.04) with a near significant effect of the predictor GSH (*B* = 0.99, *p* = 0.06).

**Table 3:** Neurometabolite predictors of heavy drinking days^+^ and percentage days abstinent^+^ throughout the trial by linear regression analysis, and weighted effects with recent alcohol consumption (24 h).

**Heavy drinking days**	***R*^2^**	***F***	***B***	***p***
Model 1	0.24	2.61		0.10
GSH/Cr			−0.37	0.18
NAA/Cr			−0.17	0.53
Model 2^**^, weighted by recent alcohol consumption, *R*^2^ change = 0.61	0.85	14.28		0.01
GSH/Cr^*^			−1.22	0.01
NAA/Cr			0.41	0.23
**Percentage days abstinent**				
Model 1	0.10	0.49		0.62
GSH/Cr			0.06	0.83
NAA/Cr			0.19	0.52
Model 2^*^, weighted by recent alcohol consumption, *R*^2^ change = 0.62	0.72	6.50		0.04
GSH/Cr			0.99	0.06
NAA/Cr			−0.18	0.68

+ Calculated from the time of scanning (on average 17 days) to the end of treatment (week 12).

* Significant at p < 0.05,

** *Significant at p < 0.01, independent of treatment status. For both treatment outcomes, when considering just the patients that consumed alcohol within the last 24 h, the model yielded the highest R^2^ whereby GSH was a significant and a trend for significant predictor for heavy drinking days and percentage days abstinent, respectively*.

## Discussion

This is the first ^1^H-MRS study of the effects of baclofen on neurometabolite concentrations in alcohol dependent individuals. There were significant differences between baclofen and placebo on parietal concentrations of GSH when controlling for recent drinking, with baclofen treated participants demonstrating significantly higher levels of GSH/Cr ratio relative to placebo. That is, baclofen significantly increased depleted GSH relative to placebo for those participants that drank alcohol in the previous 24 h (although these participants were not intoxicated, reading 0.00 BAC). GSH deficiency is associated with early abstinence in alcohol dependent patients (32) and is a pathophysiological characteristic of alcohol-related hepatotoxicity (33). We also observed that decreased GSH/Cr predicted greater heavy drinking days throughout the trial following the scan and increased GSH/Cr predicted greater percentage days abstinent throughout the trial. These results support a growing number of preclinical studies and one clinical study suggesting that replenishing intracellular levels of GSH may play a role in reducing alcohol withdrawal (34), alcohol consumption (35) and relapse (36). These results are also consistent with our previous published data demonstrating accurate low levels of GSH with an *R*^2^ = 0.98 using short TE PRESS sequence based on the cysteine moiety i.e., (7CH_2_) at 2.97 ppm (37), despite suggestion in the literature that a short TE PRESS sequence is not capable of accurately resolving low concentrations of GSH (38).

GABA_B_ mediated signaling has previously been suggested to play a role in oxidative stress. For example, baclofen has been found to attenuate oxidative damage and neuroinflammation that is induced by MPTP in rats (39), reduce stress-induced brain NO_*x*_^−^ levels in rats (40) and has also been found to be neuroprotective by reversing oxidative stress and free radical damage *in vitro* (41). Moreover, administration of GABA has been reported to reduce the level of oxidative stress markers in the rat brain following streptozocin-induced (STZ) oxidative stress (42). One study observed a GABA_B_ antagonist but not high dose baclofen reversed reduced GSH following STZ (43). Thus, there may be some GABA_B_ mediation of oxidative stress yet the exact nature of this relationship remains to be determined. The deficit in GABAergic signaling that may occur in alcohol dependence has been implicated in the up-regulation of glutamatergic excitotoxic neuronal damage which may also lead to oxidative stress (44). Low dose agonism following baclofen may enhance GABA release which in turn regulates excitotoxic signals and subsequently reduces oxidative stress. Further, preclinical studies have demonstrated that baclofen administration decreases glutamate release (45) and a direct relationship has been observed to occur between microglial glutamate uptake and GSH synthesis (46). However, in this study we did not observe any corresponding significant increase in parietal Glu/Cr or GABA^+^ concentrations, although this could be due to a regional disparity or limited technological sensitivity with the obtained sample size.

In the current study, we also observed significantly higher parietal levels of NAA/Cr concentrations following BAC administration relative to PL. Generally speaking, levels of NAA in various parts of the brain correlate with neuronal health or integrity, whereby decreased levels of NAA have been interpreted to indicate neuronal/axonal loss, or compromised neuronal metabolism (47). Heavy alcohol consumption has been suggested to have a disrupting influence on neuronal metabolism (13). Previous studies in alcohol dependent individuals have reported a significant negative correlation between concentrations of cortical and subcortical NAA with recent and lifetime heavy drinking levels (13, 18). Our results suggest that treatment with BAC improved compromised neuronal metabolism caused by recent drinking. It is possible that this reflects an indirect treatment effect related to increased previous abstinence due to the chronic efficacy of BAC to reduce alcohol consumption via various mechanisms. To further investigate the potential role of chronic efficacy of BAC, we examined the relationship between days on BAC and metabolite concentration. We observed a significant positive association between time on BAC treatment and NAA levels which may suggest either an indirect of beneficial treatment efficacy to reduce drinking or a cumulative effect of extended GABA_B_ mediated effects on NAA levels. Given that the BAC vs. PL acute effect on NAA and GSH was only significant in participants that recently consumed alcohol while there was no effect in patients that were abstinent, it is likely that BAC directly and acutely elevates these markers in the case of alcohol-induced deficits that occur in very early abstinence.

To this degree, it is important to note that ^1^H-MRS investigations in the literature have also revealed results that varied in relation to drinking and withdrawal status (48–50). Indeed, in the current study, the effects of baclofen on GSH/Cr and NAA levels and the predictive associations of GSH/Cr concentrations with follow-up drinking were only evident when controlling for recent alcohol consumption. Thus, our data support the hypothesis that recent alcohol consumption may account for differences in neurometabolites that may be observed in alcohol dependent patients.

There are several limitations in our study including the modest sample size, albeit not uncommon in ^1^H-MRS studies. Increased power would allow for analysis of interactions between PL vs. BAC, abstinence vs. recent drinking and the relationship between treatment outcome. Indeed, this was a secondary substudy of a larger trial and although we controlled for recent alcohol consumption and there were no differences between groups on baseline characteristics, interpretation would be improved if scanned participants were specifically randomized and all participants reported the same window of alcohol consumption. To this degree, the before vs. after treatment design with two scans scheduled at baseline and follow-up in the trial is optimal. Strengths include the inclusion of follow-up drinking outcomes in a randomized controlled trial and that this is the first examination of neurometabolites following baclofen administration in patients with alcohol dependence.

## Conclusion

This is the first ^1^H-MRS study of baclofen administration on neurometabolites in alcohol dependent individuals. Baclofen, relative to placebo, significantly increased levels of the antioxidant, GSH, and NAA a marker of neuronal integrity, in the parietal lobe of recently drinking alcohol dependent patients. In these patients, higher GSH levels predicted beneficial treatment outcomes at follow-up suggesting a role for oxidative stress in AUD. MRS is a valuable technique to study neurobiology of human AUD *in vivo* and to identify the impact of treatment.

## Author contributions

KM contributed to MRS study conception and design, supervision of the MRS study and main trial, data analysis and writing of the manuscript. JL contributed to the design of the MRS protocol and analysis. KC contributed to the MRS analysis. WL contributed to patient recruitment, conducting neuroimaging sessions and data maintenance. WL also contributed to data analysis and the presentation of the manuscript. PH and AB contributed to conception of the main trial, design and supervision. PH contributed as site investigator and physician. All authors have approved the final manuscript.

### Conflict of interest statement

The authors declare that the research was conducted in the absence of any commercial or financial relationships that could be construed as a potential conflict of interest.
